# Synthesis, characterization and antibacterial assessment of SiO_2_-hydroxypropylmethyl cellulose hybrid materials with embedded silver nanoparticles

**DOI:** 10.1080/13102818.2014.944789

**Published:** 2014-09-23

**Authors:** Tsvetelina Angelova, Nadezhda Rangelova, Hristina Dineva, Nelly Georgieva, Rudolf Müller

**Affiliations:** ^a^ Department of Biotechnology, University of Chemical Technology and Metallurgy, Sofia, Bulgaria; ^b^ Department of Fundamentals of Chemical Technology, University of Chemical Technology and Metallurgy, Sofia, Bulgaria; ^c^ Institute of Technical Biocatalysis, Hamburg University of Technology, Hamburg, Germany

**Keywords:** SiO_2_, hydroxypropylmethyl cellulose, silver nanoparticles, antibacterial activity

## Abstract

Antibacterial SiO_2_ hybrid materials based on tetraethyl orthosilicate (TEOS), hydroxypropylmethylcellulose (HPMC) and silver were prepared by the sol-gel method. The content of cellulose derivate was 5 wt% and the silver concentration varied from 0.5 wt% to 2.5 wt%. The amorphous nature, morphology and antibacterial behaviour were studied. Fourier transform infrared (FTIR) spectra of the hybrids showed characteristic peaks for SiO_2_ network. Scanning electron microscope (SEM) analysis confirmed the formation of spherically shaped silver nanoparticles with a size of 30 nm on the matrix surfaces. *Bacillus subtilis* and *Escherichia coli* K12 were used as model microorganisms. The hybrid materials demonstrated bacteriostatic and bactericidal effect on the tested bacteria. Highest sensitivity to the obtained hybrids was observed in *B. subtilis* with significant lag-phase delay and biggest inhibition zone sizes.

## Introduction

The designing and development of new hybrid materials with a wide range of industrial applications have been a main goal of many researchers in recent years and have become a promising field for life-style improvement.[1] Moreover, hybrid materials containing nanoparticles are extensively studied due to their improved properties tailored by the physical, electronic, optical and catalytic effects of the nanoparticles.[2–4] Silver nanoparticles (AgNps) are known for their broad-spectrum antibacterial activity against various strains of Gram-negative and Gram-positive bacteria and as an effective killing agent against common fungi such as *Aspergillus*, *Candida*, *Saccharomyces* and yeasts isolated from bovine mastitis.[5–11] They provide a large surface-area-to-volume ratio, which ensures better contact with microorganisms, and low resistance due to the ability of silver nanoparticles to target multiple components in the cell.[5,7,12–14] There are many assumptions regarding the mode of action underlying AgNps’ inhibitory effect on microorganisms. According to Jones and Hoek,[15] three main proposed mechanisms are known: (1) direct damage of cell membranes, (2) uptake of free silver ions followed by disruption of adenosine triphosphate (ATP) production and (iii) DNA replication and generation of reactive oxygen species (ROS) as a result of the combined effect of AgNps and silver ions.[5,16] Embedding of silver nanoparticles in a polymer or silica matrix prevents their irreversible aggregation and enhances the antimicrobial properties.[17–19] Polymer-silica hybrid materials possess a good compatibility with living-matter and when impregnated with silver nanoparticles may find application as effective biocide agents in biomedicine, water purification systems and food industry.[19–22]

In our previous investigations we reported successful preparation of silica/biopolymers/silver hybrid materials by the sol-gel method.[23–26] Hybrid materials using SiO_2_ precursors such as tetraethyl orthosilicate (TEOS) or ethyltrimethoxysilane (ETMS) and different cellulose derivatives were obtained and their structure, surface morphology and antibacterial activity were investigated. It was shown that AgNPs of different sizes and shapes were present on the hybrid surface. The homogeneity of distribution of the silver particles was additionally determined and good bactericidal activity was demonstrated.[23–25]

The main objective of the present work was to study the synthesis, structure and properties of new amorphous hybrid materials based on SiO_2_ and hydroxypropylmethyl cellulose prepared by the sol-gel method. In this context we checked the incorporation effect of silver ions and the assessment of their antimicrobial properties in the new materials.

## Materials and Methods

### Chemicals and reagents

Hydroxypropylmethyl cellulose (HPMC), tetraethylortho silicate (TEOS) and silver nitrate (AgNO_3_) from Sigma–Aldrich® and nitric acid from Merck (a. g.) were used without any further purification.

### Microorganisms

All strains used in the present study were obtained from the teaching laboratory of the Hamburg University of Technology (Hamburg, Germany).

### Preparation of SiO_2_/HPMC/silver hybrid materials

The sol-gel method was used for preparation of the new hybrids by acid catalysed hydrolysis. The silica precursor was pre-hydrolysed with H_2_O and 0.1 mol L^−1^ HNO_3_ solution and different amounts of AgNO_3_ were added to this sol under stirring. The content of silver was varied from 0.5 wt% to 2.5 wt%. HPMC dissolved in H_2_O (5 wt%) was mixed with the above-mentioned silica-silver sols. For gelation, ageing and drying processes the obtained sols were isothermally treated at 50 °C.

### Characterization methods

Infrared spectra of solid samples were recorded on a Bruker's VERTEX 70 spectrometer. X-ray diffraction (XRD) patterns were used to investigate the crystallinity of the materials. For the XRD measurements a Bruker D8 Advance diffractometer was used at Cu Kα radiation in the range of 10 < 2*θ* < 60. X-ray energy dispersive spectrometry (EDS) was performed using an iridium X-ray fluorescence (IXRF) system integrated to a Gemini LEO 1530 scanning electron microscope (SEM) operating at 10 kV. The investigated samples were covered with gold, using BAL-TEC/SCD 050 Sputter coater. The optical density (OD) was measured by a Libra S12, Biochrom Ltd spectrophotometer.

### Effect of SiO_2_/HPMC/Ag hybrids on bacterial growth

Two different methods were applied to confirm the antibacterial activity of the SiO_2_/HPMC/Ag hybrids.

### Measurement of the optical density of the culture in the presence of antibacterial materials

To investigate the bactericidal properties of the materials obtained regarding *Escherichia coli* K12 and *Bacillus subtilis*, the overnight pure cultures were prepared by loop-inoculation in 50 mL liquid Luria–Miller (LB) medium. These primary hybrid materials were pre-coated onto cover slips and dried at 50 °C. Aliquots (100 μL) of bacterial suspension were carefully spread on cover slips with different concentration of silver (0.5 wt%, 1.0 wt%, 1.5 wt%, 2.0 wt% Ag) and incubated for 1 h. Sterile flasks with 30 mL of LB medium were supplemented with the incubated dry cover slips. The positive control contained silver-free materials and bacteria. The cultivation process was performed on a rotary shaker (120 r min^−1^) at 37 °C for *E. coli* K12 and 30 °C for *B. subtilis* for 30 h. Growth rate and bacterial concentrations were determined by OD measurements at 600 nm.

### Agar diffusion test

The potential antibacterial activity of the hybrids against *E. coli* K12 and *B. subtilis* was evaluated on the basis of the inhibition-zone size. Aliquots (350 μL) of *E. coli* K12 and *B. subtilis* suspension diluted 10-fold were seeded on agar plates with solid LB medium by the pour plate technique. After 10 min, cover slips pre-coated with the hybrid materials were added to all plates. Each of the samples corresponded to different silver concentrations (0.5 wt.%, 1 wt.%, 1.5 wt.%, 2 wt.% Ag) and each plate contained three replicates of samples. The size of the inhibition zones was measured under a microscope after 12 h of incubation.

## Results and discussion

**Figure 1.**
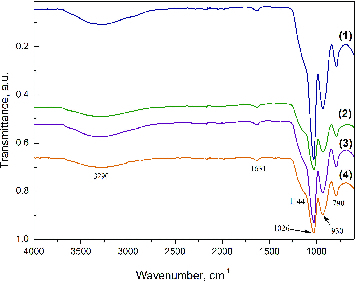
Infrared spectra of hybrids derived with different silver content: 0.0 wt% Ag (1), 0.5 wt% Ag (2), 1.5 wt% Ag (3) and 2.5 wt% Ag (4).

The infrared spectra of SiO_2_/HPMC/Ag hybrid materials with different Ag content are shown in Figure 1. The bands in the range from 3200 to 3600 cm^−1^ can be attributed to contribution of a silanol (Si–O) stretch and absorbed water on the surface.[27–29] The vibration of silanol groups which are associated with molecular water through H-bonds can be found above 3500 cm^−1^.[30] There are reports [29–31] that the bands over 3500 cm^−1^ can be attributed to the vibration of hydrogen bonds between the organic and inorganic components in the hybrid materials. The bands at ∼1630 cm^−1^ can be attributed to the vibration of a small amount of absorbed water.[32] In the area between 1300 and 600 cm^−1^ the characteristic bands of the silica network are found. The bands at about 1080 and 790 cm^−1^ can be assigned to the asymmetric and symmetric vibrations of the siloxane linkage (Si–O–Si).[27,29–31] The bands at 950 cm^−1^ can be attributed to the silanol (Si–O) stretch. The shoulder at (1000–1200) cm^−1^ is typical for the Si–O–Si asymmetric stretch.[29–31]

**Figure 2.**
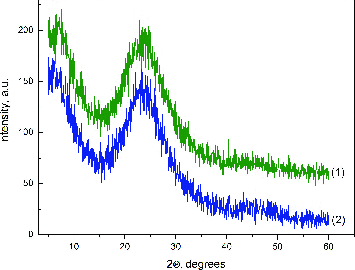
XRD patterns of hybrids derived with different silver content: 0.5 wt% Ag (1) and 2.5 wt% Ag (2).

Figure 2 shows the XRD patterns of hybrid materials. The diffractograms convincingly showed the amorphous nature of the obtained hybrids. The absence of silver diffraction peaks in the samples can be explained by the overlap of a very broad peak of silica with the silver peaks, which were present in too small trace amounts to be detected by XRD.[33,34]

**Figure 3.**
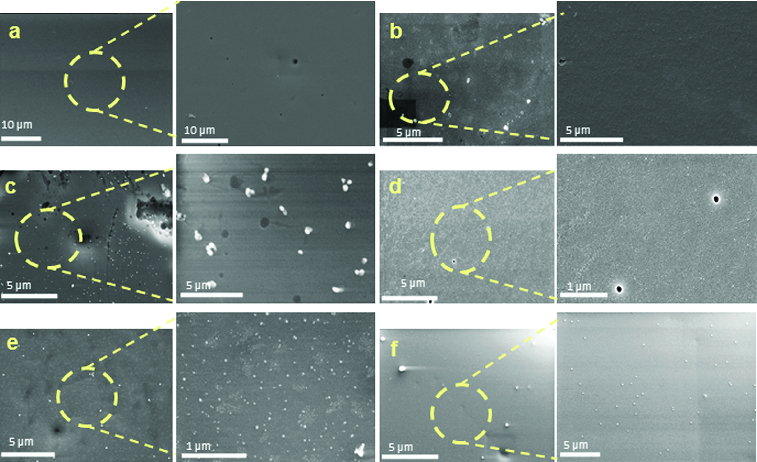
SEM images of hybrids derived with different silver content: 0.0 wt% Ag (a); 0.5 wt% Ag (b); 1.0 wt% Ag (c); 1.5 wt% Ag (d); 2.0 w% Ag (e) and 2.5 wt% Ag (f), at different magnification.

Figure 3 shows scanning electron microscopy images of the obtained silica hybrids. The images revealed that the surface of the sample without silver (Figure 3(a)) is completely smooth and homogeneous in morphology. As seen from the image made at higher magnification, completely different morphology was observed for the hybrids obtained with a different silver content. The sample containing 0.5 wt% silver had a smooth surface with wavy character (Figure 3(b)). Silver nanoparticles with spherical shape and size of 30 nm and clusters unevenly distributed on the sample surface were observed with 1.0 wt% Ag (Figure 3(c)). Increasing the silver concentration in the matrices up to 2.5 wt% Ag led to their more uniform distribution and significant reduction of silver agglomerates; as a result single nanoparticles became predominant on the sample surface (Figure 3(d)–(f)).

**Figure 4.**
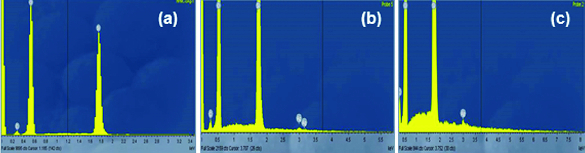
EDS analysis of hybrids derived with different silver content: 0.0 wt% Ag (a), 1.0 wt% Ag (b) and 2.5 wt% Ag (c).

The chemical composition of the samples was determined by EDS (Figure 4). The spectrum clearly showed the presence of a highly intensive peak at ∼1.7 keV, corresponding to Si. The peaks for О and С were also detected. The results for the SiO_2_/HPMC/Ag hybrids showеd that increasing the Ag contents led to an increase in the intensity of the peak at about 3 keV on the hybrid surface.

**Figure 5.**
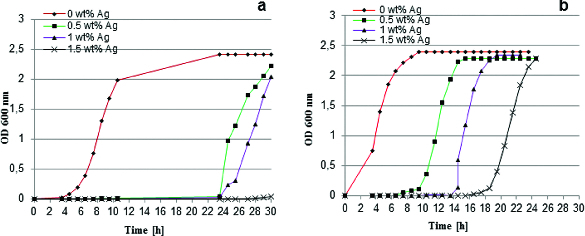
Bacterial growth curves for *B. subtilis* (a) and *E. coli* K12 (b) in the presence of hybrids derived with different silver content.

The antibacterial potential of the obtained hybrid materials was investigated by studying the bacterial growth of two reference strains in the presence of 0 wt%, 0.5 wt%, 1 wt% and 1.5 wt% Ag-containing materials in batch culture. *E. coli* K12 and *B. subtilis* were used as model representatives for Gram-negative bacteria and for Gram-positive bacteria. The silver-doped hybrids had a growth-restraining effect on both microorganisms and resulted in diverse lag phase delays, whereas the growth rate remained unchanged (Figure 5). *B. subtilis* was more influenced by the studied materials with significant lag delays up to 20 h (Figure 5(a)) as compared to *E. coli*, which had only 6 h prolongation of the lag phase (Figure 5(b)). The bacteriostatic effect of the materials was dependent on the silver concentration. The growth of the more sensitive *B. subtilis* exposed to materials with 1.5 wt% Ag content was completely inhibited during the study period (Figure 5(a)).

**Figure 6.**
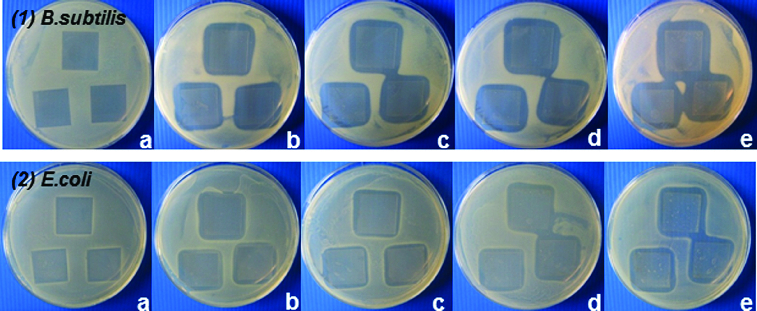
Inhibition zones of *B. subtilis* (1) and *E. coli* K12 (2) exposed to pre-coated and dried cover slips with hybrids derived with different silver content: 0.0 wt% Ag (a), 0.5 wt% Ag (b), 1 wt% Ag (c), 1.5 wt% Ag (d) and 2 wt% Ag (e).

As a second endpoint, the antibacterial effect of the studied SiO_2_/HPMC/Ag hybrid materials against Gram-negative and Gram-positive bacteria was additionally assayed by measuring the inhibition zones formed around the materials containing 0 wt%, 0.5 wt%, 1.0 wt%, 1.5 wt% and 2.0 wt% Ag. The test results revealed well-formed zones free of growth around the silver-doped matrices. The size of the inhibition zones (respectively, the antibacterial effect) was silver-concentration dependent (Figure 6).

The results obtained by this method also showed the Gram-positive bacterium *B. subtilis* to be more sensitive, as the zones of inhibition increased from 3.22 to 4.58 mm with increasing the silver concentration. In comparison, the zones of inhibition for *E. coli* K12 were smaller than those for *B. subtilis* at the same silver concentrations (Figure 6, Table 1). These results can be explained by the differences in the cell walls of the strains, as it is well known that Gram-negative bacteria possess an outer membrane lacking in Gram-positive microorganisms that covers the peptidoglycan layer. The essential function of the outer membrane is to serve as a selective permeability barrier protecting bacteria from harmful agents, such as detergents, drugs, toxins and degradative enzymes, but also penetrable to nutrients sustaining bacterial growth.[35]

**Table 1.** Size of inhibition zones (mm) surrounding SiO_2_/HPMC/Ag hybrids.

SiO_2_/HPMC/Аg hybrids	0.5 wt%	1.0 wt%	1.5 wt%	2.0 wt%
Microorganism				
*Bacillus subtilis*	3.22 ± 0.18	3.79 ± 0.11	4.13 ± 0.10	4.58 ± 0.16
*Escherichia coli* K12	1.42 ± 0.2	2.19 ± 0.30	2.55 ± 0.32	2.84 ± 0.30

Note: Data are means of three replicates *±* SD (standard deviation).

## Conclusions

In the present work we prepared hybrid materials based on SiO_2_ and hydroxypropylmethyl cellulose with embedded silver nanoparticles. Investigation of their potential as bacteriostatic agents was performed. The XRD patterns showed that all samples were in amorphous state. Fourier transform infrared spectra of the hybrids showed characteristic peaks for a SiO_2_ network. SEM analysis confirmed the formation of spherically shaped silver nanoparticles with a size of 30 nm on the matrix surfaces. Antibacterial properties of the hybrid matrices against *B. subtilis* and *E. coli* K12 were demonstrated. The results revealed that the *B. subtilis* strain was more sensitive because it showed slower growth in liquid media and allowed larger inhibition zones to form in comparison to *E. coli* K12. Both methods for assessment of antibacterial activity indicated that the increase in silver concentrations had an increasing inhibitory effect on the growth of microorganisms.
